# Evolution as a Solution: Franco Andrea Bonelli, Lamarck, and the Origin of Man in Early-Nineteenth-Century Italy

**DOI:** 10.1007/s10739-020-09617-2

**Published:** 2020-10-02

**Authors:** Fabio Forgione

**Affiliations:** Dipartimento di Culture e Civiltà, Università degli Studi di Verona, Viale dell’Università 4, 37129 Verona, VR Italy

**Keywords:** Evolution, Lamarckian transformism, Jean-Baptiste Lamarck, Franco Andrea Bonelli, Origin of man, Italy, Nineteenth century

## Abstract

Franco Andrea Bonelli, a disciple of Lamarck, was one of the few naturalists who taught and disseminated transformism in Italy in the early nineteenth century. The explanation of the history of life on Earth offered by Lamarck’s theory was at odds with the Genesis narrative, while the issue of man’s place in nature raised heated debates. Bonelli sought to reconcile science and religion through his original interpretation of the variability of species, but he also focused on anthropological subjects. Following Blumenbach’s studies, he investigated the differences within the human species and explored the topic of humans’ alleged superiority over animals. The origin of human beings, their history, and their relationship with the rest of life were thus read in light of transformism. According to Bonelli, such questions would not do irreparable damage to the new theory. On the contrary, he considered evolution not only acceptable but even necessary to protect the Holy Texts from certain dangerous trends in anthropological and zoological research.

Due to political fragmentation and the weakness of scientific institutions at the beginning of the nineteenth century, Italian natural history scholars took a great interest in the activities of the leading European research centers. This was also the case in Piedmont, a region that had been ruled for centuries by the Savoy dynasty and was historically linked to France by many geographical and cultural ties. Unlike other Italian territories, Piedmont did not become part of the Kingdom of Italy—a vassal state of the Napoleonic Empire—but was instead directly annexed to France and placed under imperial administration. Thus, Paris became both a political point of reference and a privileged scientific pole of attraction for Piedmontese naturalists. Undertaking a challenging journey through the mountains, many scholars moved to the transalpine capital, where they had the opportunity to study museum collections that were far more organized and comprehensive than those available in Italy, as well as to meet leading scientists of the time. This was the path taken by Franco Andrea Bonelli (1784–1830), who distinguished himself as a solid supporter of Lamarck’s theories on the variability of species. In Italy, transformism was widely circulated, though it was not central to scientific debate and was often confined to private reflections (Corsi 1984). Although Bonelli is no exception, as we will see, he nevertheless provides a rare example of the presence of Lamarckian theses in the Italian academic panorama. Bonelli’s anthropological work is another factor that renders him so interesting, testifying to an early responsiveness to European scientific research. These two lines of investigation were inevitably destined to intertwine, providing innovative interpretations of human history.^1^

## Bonelli’s Early Years

Franco Andrea Bonelli was the twelfth son of Tommaso and Veronica Boschis. He was born in Cuneo on 10 November 1784, not long before the end of the *ancien régime*, sealed by the surrender of the Kingdom of Sardinia to the French in December 1798. Coming from a well-off family, Bonelli began his education at the College of the Somascan Fathers in Fossano at the age of ten, before moving to Turin with his father. According to his biographers, in these years his interests centered on mechanics, but this soon gave way to natural history and, in particular, ornithology—perhaps because of the similarity between flying machines and bird wings.^2^

Bonelli began to read the key texts on natural history available to him and to spend time with some of the naturalists working in Turin.^3^ Among them were Michele Spirito Giorna (1741–1809), who had taught zoology and comparative anatomy at the local university since 1802, the anatomist Luigi Rolando (1773–1831), and the physician Giambattista Rubinetti (1776?–1827), an amateur ornithologist and entomologist. Through exchanges and excursions with his friends, Bonelli began to assemble a collection of birds and insects, which was also visibly enriched through his correspondence with Italian and European naturalists.

While ornithology was Bonelli’s first passion, this was followed by entomology, as demonstrated by the twenty-two zoological and six hunting notebooks he drafted in the early nineteenth century. Initially devised as a collection of tips for bird hunting, with a view towards a work Bonelli planned to publish, these notebooks assumed a different character over the years, coming to take the form of an almost daily diary of observations, scientific descriptions, and curiosities, as well as of interesting reflections on taxonomic issues. In these notes—dating from the time Bonelli was between fifteen and twenty years of age—as well as in his correspondence with the Genoese entomologist Massimiliano Spinola (1780–1857), one can detect a number of questions that would leave their mark on all his work. A careful observation of anatomical differences led Bonelli to explore the limits of species and of constant varieties that, in his opinion, were the result of the action of “climate or nourishment.”^4^ No matter how convinced he was of the wisdom of nature and its overall harmony, Bonelli was already sensitive to the question of the variability of species owing to environmental factors. Thus, in the early months of 1807, he noted that the shape of organs seemed to be the consequence of life habits and not vice versa:

The organs seem to be intended for the way of life, not the way of life for the organs; evidence of this can be found in ducks. The wild duck, which spends its life in lakes and rivers, has compressed legs, as do all swimming birds; the legs of the domestic duck, on the contrary, which is forced to live in a farmyard, where it can finds only a little water but not enough to swim in, are almost round. Other parts such as wings, membranes etc. must also be subject to changes. Rarely do we see a domestic duck fly.^5^

Lamarck held similar views on the subject, but examples of adaptive morphological features in birds—often associated with teleological considerations—were also common in other contemporary works. Bonelli was certainly familiar with the *Nouveau dictionnaire d’histoire naturelle* (1803–1804), one of the great encyclopedic dictionaries published in France, which was edited by Julien-Joseph Virey (1775–1846), a physician and disciple of Buffon. Virey wrote several entries for the dictionary and quoted extensively—albeit critically—from Lamarck’s studies, thus facilitating their circulation.^6^

During this period, Bonelli also looked into a new method of classification, which would move beyond the unsatisfactory chain of beings propounded by Charles Bonnet (1720–1793) and provide a more accurate reflection of the complex interactions among living beings. He, therefore, began to develop alternative representations in the shape of concentric circles or networks.^7^

This youthful apprenticeship took shape in a short publication completed in 1807, entitled *Specimen Faunae Subalpinae*, in which Bonelli described thirty species of Piedmontese beetles (Bonelli 1812). Shortly afterward, he presented another work on the same subject at the Academy of Sciences in Turin: *Observations entomologiques* (1811a, 1813). In the meantime, he also wrote a *Catalogue des oiseaux du Piémont* (1811b), which set out the Latin, French, and Piedmontese nomenclature of over 200 species of birds. These were the only printed works by Bonelli, apart from a few other zoological memoirs; many of his handwritten notes were destined to remain unpublished.

In May 1809, a chair at the Academy of Sciences became vacant upon the death of Michele Spirito Giorna. Thanks to the esteem he had earned through his *Observations entomologiques*, Bonelli was chosen as Giorna’s successor, thereby becoming a full member of the subalpine scientific elite. In addition, Giorna’s position as Chair of Zoology at the university was open. The distinguished French naturalist Georges Cuvier (1769–1832), an advisor for the Imperial University at the time, was charged with finding a successor. Between 1809 and 1810, Cuvier traveled through Italian departments, inspecting the existing schools and universities to align them with French institutions. In Piedmont, Cuvier found the university to be advanced and efficient, which was the legacy of the “well-administered” Savoyard state.^8^ A few years earlier, Napoleon himself had been impressed by the centralization of the Piedmontese educational system, taking it as a model for reorganizing the entire Imperial University. During his time in Turin in March 1810, Cuvier got to know Bonelli; he was impressed by his work and suggested that he expand his knowledge with a view toward being appointed to the chair of zoology. He invited Bonelli to visit Paris and the *Muséum national d’histoire naturelle*, which was the hub of European zoological studies at the time, where foremost scholars of the continent would meet.

Anxious to prove himself up to the task, in July 1810 Bonelli left Turin for Paris, where he took courses at the *Muséum* and continued his research on beetles. This one-year stay in the imperial capital was marked by his final acceptance of the transformist theory, which he would go on to develop and defend throughout his career. A key moment in this process was his meeting with Jean-Baptiste Lamarck (1744–1829), professor of invertebrate zoology, who took an immediate interest in and liking for the young Piedmontese scholar. Bonelli was, in fact, familiar with the Lamarckian theses even before his trip to Paris: the pair could then engage with one other on common ground since they shared the same fundamental ideas on the variability of nature.^9^ Bonelli was one of the twenty-five Italian students of Lamarck and one of the very few to disseminate his ideas in Italy, together with the Neapolitan Giosuè Sangiovanni (1776–1849).^10^

## Bonelli and the Transformist Theory: The Letter to Franz Ziegler

Once he had returned to Turin in the autumn of 1811, Bonelli settled into the position of Chair of Zoology, which he occupied until his death in 1830, and began to offer courses that included a considerable focus on transformism. Evidence of his beliefs can be found in his preparatory notes for his lectures and in the handwritten “philosophical” reflections published at the beginning of the twentieth century by the zoologist Lorenzo Camerano (1856–1917).

One particularly interesting source can be found in a letter that Bonelli sent in January 1813 to his colleague Franz Andreas Ziegler (1761–1842), custodian of the Vienna imperial-royal cabinet of natural history. Bonelli had been in contact with Ziegler since 1806, when he was working in the entomological section of the cabinet.^11^ The letter from 1813 is significant; it provides a clear synthesis of Bonelli’s positions, his attitude towards Lamarck, and the anxieties that the support for certain ideas could elicit at the time. Given his ideas on the variability of species, Bonelli highlighted the existence of a link between two beetles, *Carabus cyaneus* and *Carabus coelatus*, arguing that the former might have originally been a variety of the latter. Ziegler disagreed and in March 1812 wrote a letter to criticize his colleague’s view. Bonelli returned to the matter a year later, when, to explain this minor question, he broadened the discussion to include a wide overview of his ideas on nature.^12^

At the beginning of his letter, Bonelli unhesitatingly branded the constancy of nature and final causes as “prejudices,” thereby disrupting the Aristotelian legacy that continued to dominate natural history. For him, such convictions were merely the result of religious beliefs that had not been adequately contested or of an almost childish astonishment at the wonders and order of nature. Such order and constancy were merely transitory, such perfection was merely superficial: they were the result of the apparent impossibility of explaining natural phenomena using the tools of science. Therefore, it was necessary to move beyond established opinions and to give due consideration to certain unquestionable facts, such as the differences between the races of human beings, dogs, or other domestic animals. Such variations were not due to captivity itself, but rather to the many combinations of climate, nourishment, and habits.

The way organisms were conditioned by their *milieu*—the very essence of Lamarck’s theory—could be proved a posteriori by the study of birds, animals Bonelli knew well. Their plan of organization usually consisted of four fingers—three that turned forward as well as a thumb that turned backward to allow them to rest on branches. However, thumbs proved useless in species that lived on the ground or in water; as a matter of fact, they appeared to be less developed and even on the verge of disappearing, as in the genus *Charadrius*. Bonelli saw this as confirmation of the absence of final causes: a wise nature would have ruled out the thumb completely from the start, while the descending gradation could only be explained by the progressive transformation of living beings according to external circumstances.

An in-depth examination of the nature of such circumstances and how they could have acted on the organism was still required. In general, however, two processes could be observed in nature: a tendency towards perfection or increased complexity and an adaptation of organs to habits according to the theory of use and non-use. While acknowledging Lamarck’s contribution to the study of these phenomena, Bonelli did not hesitate to offer harsh criticism for his excessive speculation:

In his *Philosophie zoologique*, Mr. Lamarck presented (albeit only after many observations by Pallas, Buffon and many other observational zoologists) germs and a great deal of indirect evidence in favor of such a doctrine. Yet if the author himself had been less a thinker and more a meticulous observer, and if, most importantly, he had gone into the minute details of some aspects of zoology and studied a larger number of animals, he would somehow have avoided certain mistakes and many ridiculous considerations that inflict great damage upon the well-founded observations and considerations with which they are mixed up.^13^

An equivalent for the well-known variations among domestic animals could be found in wild animals and their varieties, which were usually believed to be constant if examined in over a short period of time and under stable circumstances. Furthermore, the ambiguity of the notions of species and variety led zoologists to be overzealous and create a new species for each slight variation. According to Bonelli, who claimed that only individuals existed, the advancement of science would prove traditional taxonomic divisions to be groundless.

Circumstances could vary according to infinite and obscure combinations, even in very narrow spaces, resulting in the most diverse animals; nevertheless, *susceptibilité*—namely, adaptation to the environment—was just one of two factors involved in the transformation of species. Following Lamarck’s idea of the *marche de la nature* [march of nature], Bonelli also believed in a tendency towards perfection—named *développement*—which acted both at the level of the individual and at higher taxonomic levels.^14^ While individual growth was easily detectable, the development of a whole species occurred more slowly, “like a clock sphere that turns every thousand years, and whose motion thus becomes visible and noticeable to us only by comparison made in distant times.”^15^ Such a movement could only be perceived by considering the different degrees of organization of the classes occupying the rungs of the evolutionary ladder.

Lamarck had introduced the notion of *pouvoir de la vie* [power of life] or *marche de la nature* in his *Recherches sur l’organisation des corps vivans* (1802) and supported it with increasing insistence in his later works. Bonelli’s distinction between *développement* and *susceptibilité* followed the fundamental lines of Lamarck’s argument but lacked the theoretical support Lamarck found in his physicochemical tenets. Despite his inconsistent statements (which have sparked intense debates on the relative value of the power of life or, rather, the effect of circumstances), scholars agree that in Lamarck’s case, “la cause qui tend sans cesse à composer l’organisation” [the cause that constantly tends to complicate organization] had no vitalistic value.^16^ Far from it; the idea of the power of life was inextricably bound up with the role of circumstances and hinged on the plastic properties of organic movement, a fundamental attribute of vital phenomena.

According to this view, bodily fluids, by forging paths and channels in the organism, could increase its size, complicate its structure, create new organs, and render their functions more specialized.^17^ These movements were closely related to use and disuse, therefore dependent on circumstances, and explained both ontogeny and phylogeny.^18^ The growth of a single organism from the embryonic to the adult state was due to *mouvement organique* [organic movement], but its repetition over long periods of time, combined with heredity, had also resulted in the gradual complexification of the animal series.^19^ Bonelli placed a strong emphasis on this symmetry, but his analysis proved less refined, given that it lacked any reference to fluid physics and organic movement. As a result, Lamarck’s mechanism and his rejection of vitalistic or teleological principles could also appear to be undermined. While on the one hand Bonelli firmly rejected the idea of final causes, he nonetheless admitted, albeit hypothetically, divine intervention to explain *développement* or described it as a random phenomenon or unintelligible force.^20^

Lamarck had used the notion of the *marche de la nature* to account for the increasing complexity that could be noticed when comparing animal classes, the so-called *grandes masses* [great groups] identified through the study of the main organic systems. Local and varying circumstances explained the external characters of species and the deviations of the animal series.^21^ Bonelli followed this pattern, associating development with the “gradation des êtres et leur enchaînement” [gradations of beings and their connections] and susceptibility with the “branches et la variété” [branches and variety].^22^ For Lamarck, however, the march of nature was not truly autonomous: it was an artifice that was useful for underlining the factual reality of a graduated animal series, but could not be separated from the dynamic of fluids and the relationship between organism and environment. This was the essential mechanism of transformation, which ultimately also explained the increasing complexity of living beings.^23^ Bonelli, on the other hand, seemed to believe in a sharper distinction between the two factors.

This was a real turning point for Bonelli’s views, even more so than for Lamarck’s. Not only did he conjecture that a supernatural intervention might be at work, but he also claimed to have reconciled religion and science through his interpretation of the first factor of transformation. Through a personal exegesis of the Bible, he justified the march of nature through God’s command *crescite et multiplicamini*: “multiply” was a clear reference to reproduction, while *crescite*—literally “grow”—must stand for something other than individual growth, since all animals had been created as adults. It was here that Bonelli found a point of contact with his theories, interpreting *crescite* as an invitation to increase the number of varieties and species, that is, to transform.^24^

Later in his letter to Ziegler, Bonelli avoided the objection that had already been pitted against transformism after the discovery of mummified animals during Napoleon’s Egyptian campaign. Cuvier had indeed found a perfect similarity between mummies and existing animals, which he had taken as the basis for an argument in favor of the fixity of species. As Lamarck had done in *Philosophie zoologique* (1809, vol. 1, pp. 69–71), Bonelli replied that the elapsed time was too short to produce variations, especially in stable environmental conditions. The mummies found in the Egyptian tombs, however, were humans or animals with an already “perfected” organization, which would therefore not show traces of the natural tendency towards perfection.

Writing with a transformist slant—or even simply expressing such convictions—was imprudent for various reasons, however. Bonelli asked Ziegler to return his letter after reading it carefully and reflecting on its contents. This paved the way for a reflection on social and religious questions, which help explain his caution and the absence of works devoted to a topic that was always at the center of his research. Bonelli believed that early-nineteenth-century Piedmont was not ready to acknowledge and accept such theories, which went beyond the methods with which natural history had been developed up to that point. It would be even less so after the Restoration, just a few months later.

The variability of species was on a collision course with divine creation. Bonelli confessed to Ziegler that he was “intimately convinced of the existence and omnipotence of a God” who had brought the world to life and given beings the faculty to develop. However, he branded the idea of a Supreme Authority that enjoyed creating infinite varieties that were completely similar to each other, except for tiny differences, as “ridiculous and childish.” According to Bonelli, attributing such *trifles* to the work of a higher will was:

as unworthy of a reasonable man as wishing to still believe that the sun revolves around the earth would be, that *all* the animals *– indistinctly –* were in Noah’s Ark, and other similar childish, inconsistent, and foolishly imagined nursery rhymes believed only by those born blind, those lacking any common sense, or by those who at least intend to make no use of it.^25^

Despite his inherent religious convictions, Bonelli was sure that revealing his ideas would lead to accusations of rashness and disbelief, placing him in an uncomfortable position against the social order.

Thus far, we have considered the personal and religious reasons for Bonelli’s silence, but there was also a problem related to scientific method. The continuous, unlimited transformation of nature threatened all the taxonomic efforts carried out by naturalists. These ultimately proved useless, since the natural productions they claimed to define were destined to vary. A transformist zoologist had little interest in a natural history made up only of labels, and Bonelli maintained that he was simply sticking to his principles when he suggested that the *Carabus coelatus* could be a variety of the *cyaneus*. A classification that established a barrier between these two beetles while being content to consider every breed of dog as belonging to the same species, even if they differed greatly in appearance, was incongruent.

In short, two very dissimilar ways of approaching the discipline existed: that of the systematic naturalist, who judged animals based on their current anatomical characters (which were accorded a decisive value since they were considered to be stable and real), and that of the philosopher naturalist, who was eager to investigate the changes animals had undergone over the centuries, along with their origin and genealogy. The work of the practical zoologists was, at first sight, useful and coherent, because the two mechanisms of transformation only acted over very long timespans. However, if one reflected on this immense time scale, there was no doubt that their taxonomic systems would prove wrong. Bonelli conceded that a practical naturalist could—and indeed should—take no interest in philosophical issues that would “soon plunge his work into chaos,” depriving him of the reassuring compass provided by so-called constant characters. To a certain extent, to continue to “produce heavy, classic books of natural history,” one had to resign oneself to using traditional methods.

In the final part of his letter to Ziegler, Bonelli proposed another argument, which touched on the field of anthropology. Earlier, Bonelli had pointed out that dogs were all generally considered to belong to the same species, despite their vastly different appearances. He then proceeded to look at the similar case of the human species:

Man, for example, is white here, black in Africa, short in Lapland, tall in Patagonia, and so forth; all such differences are mere bagatelles. Man is one everywhere, and while we judge him so liberally, we count the number of spots on the *24 punctata* ladybug and the *Tinea evonimella*, we observe the different changing shades of a butterfly, we separate the *lucanus capreolus* from the *cervus*, we distinguish the hare from the rabbit, the *cicindela danubialis* from the *hibryda* etc., etc.^26^

Bonelli was taking a position in the debate on the descent of the human races and standing up for the theory of monogenism, according to which all races shared a common origin. The problem of man’s place in nature remained a focal question in the struggle against evolutionary theories for a long time, up to the *Descent of Man* (Darwin 1871) and beyond.

## The Classification of Man

The remarks Bonelli slipped into his letter to Ziegler, almost in passing, were the result of reflections he had been engaged in over the years, revealing an anthropological interest that was still rare among Italian naturalists. Aware of how sensitive a subject the question of man was, Bonelli overturned it in favor of transformism and used it as a solution to religious concerns.

At the time, the natural history of man was no longer a novelty. Since the middle of the eighteenth century, European naturalists had included human beings—as a species—in the scope of their discipline, classifying them in relation to other animals and in light of the physical differences observed in the various regions of the globe. In their exploration of the boundaries between man and apes, scholars had to reckon with a long tradition of fabulous accounts, pathological cases, and intermediate forms, and the question was closely linked to the understanding of the differences within the human species.^27^ As Duvernay-Bolens (1995) noted, there was a relationship between the place assigned to man in the animal kingdom and the classification of human varieties or races. Between the eighteenth and nineteenth centuries, the advocates of two opposing lines of thought confronted each other, namely the monogenists—who believed in a common origin of all men—and the polygenists—who, on the contrary, maintained that distinct stocks and multiple human species existed. An analysis of their positions reveals a correlation between the taxonomic level at which man was placed and his internal subdivisions: the closer man came to apes, the more a disruptive tendency emerged within the human species, which was then divided into multiple races or species. The bitter confrontation between these factions led to the emergence of a “science of races” (Blanckaert 2003), which relegated the question of the origins of man to the background and instead placed the classification of existing groups at the center of attention.^28^ Before delving deeper into Bonelli’s ideas about man, it is worth briefly outlining the main anthropological concepts that had emerged in previous decades.

Carl Linnaeus (1707–1778) claimed that, as a historian following the principles of natural science, he could find no difference between man and monkeys.^29^ In the tenth edition of *Systema Naturae*, he placed the genus *Homo* in the mammalian order of primates—alongside *Simia*, *Lemur*, and *Vespertilio*—and divided it into two species: *Homo sapiens* and *Homo troglodytes*. Yet Linnaeus was still employing a purely logical-classificatory framework, and this division did not affect his unitary vision of man.^30^

Linnaeus’s solution was contrasted with the fact that man, the only two-handed being, belonged to the distinct and autonomous order Bimana. Georges-Louis Leclerc, count de Buffon (1707–1788) was the first to use this label, while remaining convinced that, as a natural object, man should be described and placed among animals. Anatomically speaking, there was no radical discontinuity, but, as Linnaeus maintained, the dignity of man emerged strongly at the moral and metaphysical levels.^31^ Though Buffon was a convinced monogenist, he introduced a fully genealogical concept of race, distinct from that of variety, into the classification of man. Races were the effect of a process of degeneration from a prototype due to climatic causes and, unlike simple varieties, became constant thanks to a hereditary mechanism.^32^

Other scholars strove to find a correspondence between anatomical characters and human superiority, connecting organic structures and intellectual faculties and thus rooting the exceptionality of man in his physical forms. Among them, one key figure was the Göttingen physician Johann Friedrich Blumenbach (1752–1840), who, in his *De generis humani varietate nativa* (1795), identified and described the anatomical characters that established an impermeable barrier between man (the only genus and species of the Bimana order) and the rest of the animal kingdom.^33^ Blumenbach argued for the unity of the human species, and his theses were a fundamental theoretical basis for early-nineteenth-century monogenists, including Bonelli. However, as Claude-Olivier Doron (2016) has recently pointed out, the diachronic divide between monogenists and polygenists needs to be relativized, at least when one considers that the concept of race was not necessarily linked to the acceptance of an essentialist pattern and that it was often the monogenist authors, including through the concept of degeneration, who supported racial divisions. Even in Blumenbach’s case, the unity of the species did not imply an absolute identity of forms and was instead structured around a classification of man into five races, which gradually faded into each other and which could be discerned through craniological analysis.^34^ In his model, grounded on the notion of *Bildungstrieb*, Blumenbach recognized the Caucasian race as the undifferentiated prototype of man, from which the other races had degenerated through a deviation of their life force. His model only described physical differences, however, refusing to correlate them deterministically to a hierarchy of intellectual faculties.^35^

Craniology, the basis of Blumenbach’s work, was first theorized in detail by the Dutch anatomist Petrus Camper (1722–1789). Notwithstanding Blumenbach’s criticism of his technique, Camper exerted a significant influence on anthropological studies, since he identified the facial angle as a tool for measuring differences within the human species. Driven not only by scientific interests but also by artistic and aesthetic ones, Camper was intent on defining the characteristics of ideal beauty and determined the width of the facial angle (a hereditary feature) in various human groups.^36^ The white man was the closest to the ideal of beauty, but he was not its prototype: according to Camper, that ideal had only materialized in classical sculpture. Moreover, although the facial angle of the black races almost matched that of certain apes, he did not intend to establish any link other than of a purely geometrical and aesthetic nature and repeatedly stressed significant anatomical and physiological differences.^37^ Despite later interpretations of his theories, which were soon “disfigured” (Blanckaert 1987), there was no relation between the facial angle and the intellectual and moral faculties of human beings: after all, Camper was a convinced anti-slavery monogenist.

It was Cuvier who argued strongly that the animal organization was the root of inner faculties and that it was necessary to overcome the dualism between the body and soul and to acknowledge a link between anatomical characters and intellectual development. The French naturalist initially refused to place human aptitudes on a scale, but in an article written with his colleague Étienne Geoffroy Saint-Hilaire (1772–1844) in 1795, Cuvier identified a direct connection between the proportions of the head, the size of the brain, and its skills.^38^ The facial angle became a measure of the intelligence and the moral and cultural level of three human races, placed on a descending hierarchical scale. As a result, the black race, which showed the most ape-like traits, was considered to be stuck at a lower level of development.^39^ Thus, beyond his formal statements, and even though he considered man as the only species in the Bimana order, Cuvier came close to a “de facto polygenism” (Blanckaert 2003).

In the early nineteenth century, Lamarck contributed to such anthropological studies and did not hesitate to explain the outstanding nature of human faculties through evolutionary mechanisms. The question of man’s place in classification systems was not immediately related to transformism, but it certainly affected the understanding of man as the center of creation, as well as his origin and connection with animals. Bonelli was of course aware of Lamarck’s ideas, but his anthropological interests also led him to read the major works published between the eighteenth and nineteenth centuries. The work that struck him the most was *De generis humani varietate nativa* by Blumenbach, a polestar for anthropology scholars, but he also paid special attention to Camper’s studies.

Following the classification proposed by Blumenbach in 1795, Bonelli in his university courses listed five human varieties that gradually faded into each other: the Caucasian, the Mongolian, the Negro (or Ethiopian, to use Blumenbach’s definition), the American, and the Malaysian.^40^ The cause of the differentiation of these constant varieties or races could be found in environmental conditions: just as they had caused variations in animals, they had also affected man, at least at an intraspecific level. They had a greater effect on him because man had colonized the entire globe thanks to his ability to acclimatize. One of the most obvious variations was of course skin color and, like Blumenbach, Bonelli analyzed this extensively to determine all the possible variations produced by crossbreeding over successive generations.^41^

Blumenbach clearly stated, against any polygenic hypothesis, that all human varieties belonged to a single species. He had not accorded a properly hierarchical order to his system, limiting himself to assigning aesthetic supremacy to the Caucasian forms and features. Some years later, Bonelli, despite sharing the idea of the superiority of the Caucasian race, which he defined as “la plus parfaite” [the most perfect] or, quoting Blumenbach, as the one with a face “regardé comme le plus beau et le plus agréable” [regarded as the most beautiful and agreeable] took his distance from a position that we can now easily recognize as being steeped in Eurocentrism. After having reaffirmed the identity of all human varieties, Bonelli admitted that it was difficult to establish which was the primordial form, “since the principle of choosing the man of that subspecies that [appears] more beautiful and more regular in its forms and proportions only according to our judgment, is unreasonable.”^42^ What is more, advocating that the Caucasian race was the original one led to the others being considered as a degeneration, whereas, on the contrary, a movement of improvement was at work in nature.

Embracing Blumenbach’s conclusions, during his university lectures Bonelli took a strong position against polygenism, which, at the time, was supported by such influential figures as Julien-Joseph Virey and Jean-Baptiste Bory de Saint-Vincent (1778–1846). Virey conceived of intelligence as a subject that science could explore through its instruments and situated human races within a strongly polygenetic framework. His classification of man was widely circulated, especially thanks to the long entries he wrote for the *Nouveau dictionnaire d’histoire naturelle*.^43^ Informed by Camper’s method and by the work of the English physician Charles White (1728–1813), Virey proposed that two distinct human species existed, one with white and the other with black skin, which were further divided into races.^44^ Black men, marked by a sharper facial angle and brutish features, were condemned to a lower degree of development, akin to that of apes and infinitely distant from the nobility of the white man.^45^

As with Virey, Bory de Saint-Vincent’s theories began to circulate through a very successful dictionary, the *Dictionnaire classique d’histoire naturelle*, and included a joint analysis of the physical and intellectual parts of the human being.^46^ Firstly, according to Linnaeus’ principles, Bory explicitly placed man among mammals and denied him a privileged position. Indeed, disagreeing with Buffon, Cuvier, and other naturalists such as Louis Daubenton (1716–1800), who had stressed the anatomical differences between man and apes, Bory placed the genus Orang alongside the genus Man within the Bimana order.^47^ According to Bory, monogenists sought every way possible to demonstrate the unity of the human species so as not to contradict the authority of the Holy Texts, hence their continued attachment to the concept of race.^48^ However, the anatomical analysis revealed strong barriers within mankind, which led Bory to distinguish as many as fifteen different species of men. The Japhetic one, namely that of the Europeans, was the most beautiful in terms of its features, while Hottentots were generally inferior in appearance and intellectual skills and demonstrated the link between the genus Man and the genus Orang.^49^

Thus far we have considered Bonelli’s classification of human races in the context of European anthropology, but what did he think about the synchronic and diachronic relationships between man and other animals? In order to clarify Bonelli’s positions on this sensitive topic, a dual distinction needs to be drawn. First, the differences between his private papers and his university lectures must be identified; and second, his study of the current differences between man and animals needs to be distinguished from his evaluation of their origin.

In his lectures on general zoology as well as in his courses on anthropology, Bonelli placed man in the Bimana order. This choice, which was in line with Buffon, Cuvier, and Lamarck’s ideas, was justified by the study of anatomy. Since a course in natural history could only consider physical aspects, Bonelli did not examine metaphysic or moral faculties, but they nevertheless had an indirect part to play. Drawing on the studies by Blumenbach, Camper, Daubenton, and Samuel Thomas Sömmerring on the position of the occipital hole, Bonelli analyzed man’s distinctive characters in depth. Anthropometry and anatomy unequivocally revealed the differences compared to other animals, including those closest to him. Quadrumans, notwithstanding their evident similarities with man, lacked those characteristics that had allowed thought and intellect to develop at the highest level. Their sensory faculties were much less acute; their hands—albeit in possession of opposable thumbs—were less ductile, and their ability to stand had been greatly exaggerated.

The conclusion drawn from the sum of these observations was clear: there were crucial differences between man and apes that fully legitimized the existence of the Bimana order. Indeed, Bonelli made the following statement at the beginning of his discussion, before adding the details we have briefly recalled:

Man by himself forms a species, a genus, an order isolated and distinct from all others, not only because of his moral faculties, but also because of some physical characters deriving from the conformation of his parts, which present the most complete, harmonic, univocal, and, finally, the most perfect combination in all respects.^50^

In light of this, man needed to be thankful to the Author of the universe, who had provided chaos with the power to differentiate and had allowed him to overtake every other animal.^51^ However, even at a superficial glance, a certain similarity undeniably existed between man and the great apes, which had prompted comparisons and analogies. In his lectures, Bonelli reserved a harsh judgment for these hypotheses, rejecting them unequivocally:

In our times, attempts have been made to find the relationships that make the ape species so similar to that of man, to find the analogy, in a word, to justify an attempt to establish a comparison between their respective organization and faculties. This idea – which is very old and which perhaps tended less to elevate man to the level that the author of nature assigned him, than to degrade him by elevating anthropomorphic animals to his level, coarse caricatures of his aspect – this idea, I say, has focused attention on several characters to which attention had not yet been paid, but which were later recognized as being of such importance, that even without these two sublime faculties – reasoning and speech – man would still be eminently distinguished and placed, without comparison, at an infinitely higher rank than apes by the sole consideration of his physical aspect.^52^

Apes were therefore relegated to the condition of “coarse caricatures” of man, but, if nothing else, the debate had the merit of stimulating new anatomical research that led to a reaffirmation of the radical otherness of man.

This comparative analysis demonstrated an unbridgeable gap that set man in a distinct, privileged position compared to other living beings. This could hold true if one remained in the sphere of natural history, avoiding the interference of morals or theology. Such a conclusion, however, was also a source of reassurance at the religious level, and Bonelli was particularly eager to reconcile science and faith. Furthermore, theses of this nature were also in keeping with the conservative atmosphere that prevailed over Piedmont after the Restoration.

## The Origin and History of Man

Bonelli was interested in studying man not only for purely descriptive purposes, but also from a historical perspective, investigating the processes that had led human beings to hold a position of supremacy. One of the murkiest questions to be examined was that of the origin of intellectual faculties in man and animals. It was on this point that Bonelli dwelled in the earliest surviving testimony of his reflections on mankind. In March 1803, he noted an interesting reflection in one of his zoological notebooks:

I think that naturalists have taken into consideration characters that are too doubtful to distinguish man from animals. They have said that man thinks, reasons, speaks. All this is worthless. First of all, man thinks. What is it to think? Is it a faculty peculiar to man? Is it a faculty dependent on his power over all other animals?^53^

The ability to think, Bonelli claimed, could not be said to be exclusive to man. Animals, too, showed a form of memory, which was precisely what constituted the core of thought. As for the ability to speak, it was evident in animals such as birds, whose faculties were in a certain sense even better than those of man, since singing allowed them to be heard and understood at great distances.

Bonelli returned to the topic of human and animal intellectual skills repeatedly, pointing out that, if there was a difference, it was a difference in degree and not in substance. In the notes entitled *Essai sur les facultés intellectuelles des animaux et sur l’origine de celles de l’homme,* written between 1812 and 1814 (Camerano 1908b, pp. 24–29), Bonelli presented various examples that confirmed animals’ ability to think. He also identified the sources of their moral faculties, which included instinct, experience, and the so-called *aptitude organique* (i.e., an anatomical structure that could be more or less favorable to processing thought). This “organic aptitude” could not be evaluated by taking man’s physical organization as a parameter of perfection: the intellectual system of birds seemed to surpass that of mammals, although their development of corresponding faculties was restricted by other factors, such as their lack of hands. From his observations, Bonelli concluded that reasoning was by no means an essential characteristic of man: all animals would likely have developed such a capacity in favorable circumstances. Like the organs of the body, therefore, even intellectual faculties were subject to a Lamarckian process of improvement.

In those years, Lamarck had also expressed similar positions, arguing that before reaching its current level of perfection, human intelligence may have emerged gradually. Since the *Recherches sur l’organisation des corps vivans*, he had pointed out a progression, not only in the organization of animals, but also in their communication skills and intelligence. Even men, who were usually the only beings to be attributed with the faculty of reason, displayed an analogous gradation, leading from a Newton or a Voltaire to the most ignorant and prejudiced individuals, who made little use of their intelligence. Like other organs, the brain—the organ of thought—could also develop its skills through continuous exercise. According to Lamarck, the remarkable divide in intelligence between man and animals could be explained by the living conditions of the latter, which simply repeated standard actions to meet their needs. There was a link between physical organization and intellect, and anatomy could have fostered the development of human faculties. Man, the only genus and species in his order, was clearly distinguished from the nearby quadrumans from his vertical station, the position of the occipital hole, and his finger mobility.^54^

So, if the differences were only at the organizational level, why not deduce that they had evolved progressively from the lower forms? Lamarck thus introduced the hypothesis of a genealogical link between man and apes. Shortly afterwards, he described the process of hominization of a chimpanzee. Lamarck’s use of the interrogative form and the future tense when dealing with these problems has raised questions about the true nature of his argument (Stoczkowski 1997), which is nonetheless consistent with his beliefs about the variability of living beings.^55^

A few years later, Lamarck took up the issue again in *Philosophie zoologique*, where he set out a detailed reconstruction of the process by which a species of quadrumans could evolve, becoming two-handed,^56^ and demonstrated how language and intellectual faculties were refined as a consequence of social life and the need to communicate complex concepts. Nevertheless, he remained cautious, presenting his explanation as a simple hypothesis, which would only have applied if man differed from other animals solely in physical respects and not also in terms of inner faculties. However, the second volume of the work confirmed Lamarck’s convictions and further emphasized the origin of intelligence and spirituality through purely physical mechanisms.^57^

As for Bonelli, some significant passages in his papers prove his belief that the mechanisms of transformation were not just an explanation of the anatomy of living beings; they also came into play with factors that were unrelated to physical organization, such as morals, the progress of civilization, and political systems.^58^ Bonelli’s ideas on the development of human faculties as a result of a process of improvement that also affected animals have been highlighted. To fully understand his views, however, it is also instructive to compare the statements in his university courses with private writings that had no editorial or educational purpose.

The lecture notes previously analyzed date from 1811 and 1813. Meanwhile, Bonelli reflected on transformism and focused on the issue of man, intending to write a book. The two-factor Lamarckian process of transformation also needed to be applied to the human species, which had developed “à la manière des animaux et des plantes” [in the same manner as animals and plants]. Man’s supremacy was the consequence of a successful combination of influential circumstances: if they had been more or less abundant, they would have produced an even more perfect being or, vice-versa, an animal like the ape. Similar mechanisms had generated elephants, hippos, whales, and parrots, which, like man, differed from the other species belonging to their orders. Naturalists could also have established special and distinct orders for them, therefore, including species as close to each other as the varieties of man.

If “rien n’a été réellement créé tel que nous le voyons” [nothing has actually been created as we see it], and if all living beings were equally affected by Lamarckian laws, then man could not be deemed to be the only exception, nor could he be taken as the outcome of a special creation: “In good philosophy we cannot acknowledge a specific creation for man, nor conditions different from those that accompanied the formation of the other inferior animals.”^59^ The study of human origins and evolution therefore overshadowed man’s uniqueness and instead underlined the reasons for his affinity with animals. The effect was an unavoidable downsizing of human ambition and anthropocentrism.

A true philosopher, impartial in his judgment of the entire mass of animals, must recognize that he alone, compared to the universe of which he believes he is the master, or at least the agent and main object, is nothing but dust, as the Holy Scripture says. It was only his pride that led him to believe the contrary. As currently observed in geology and zoology, and our knowledge about fossils, the globe existed long before man, and even all fossil animals are of earlier formation, since no human fossils have been found.^60^

Man was thus regarded merely as one living being among others, and his place in nature was relativized. The excavation of fossil deposits, which was flourishing thanks to the research of Cuvier and Alexandre Brongniart (1770–1847), seemed to demonstrate the complete absence of human remains in the deeper geological strata. According to the Genesis narrative—still widely influential, albeit no longer interpreted literally—the creation of man had taken place at the origin of the world, preceded only slightly by that of animals. Paleontology instead suggested the existence of an extremely ancient pre-human world, which called into question man’s role as the cornerstone of creation.

Bonelli did not intend to deny the superiority of man and his dominance over other animals; rather, he wished to reconsider the causes of these facts in the light of a transformist approach. It should first be noted that, beyond the effects of the two *marches* of nature, new characters often appeared because of accidental variations. They were then preserved and transmitted hereditarily only in the absence of crossbreeding with individuals that did not possess them. This mechanism explained, on one hand, the perfection of man and his belonging to a single species and, on the other, the impossibility of animals reaching this level and the increasing number of animal species.

Among wild animals, mating occurred freely, while human laws generally forbade marriages between kinsmen. This social convention led to a continuous crossing of different families and the inevitable elimination of accidental variations. Man was therefore affected only by the slow and continuous action of *développement* and by the conditioning of local circumstances. Environmental factors certainly rendered human races dynamic and modifiable, but, due to the great distances between one climatic zone and another, crossbreeding between different varieties was rare. As such, the individual characteristics of each race were maintained and their peculiarities increased. However, all races were bound by intermediate rings because of the gradual transitions from one climate to another, which thus preserved their unity.

Once accidental variations had been discarded, the effect of the tendency towards perfection could freely develop, widening the gap with animals evermore. In the latter, the sum of accidental variations slowed down development: instead of a “vertical” improvement, a “horizontal” multiplication of the number of races and species occurred. Yet man himself was a factor—and one of no little importance—that conditioned the perfection of animals. As soon as he had “taken up the scepter of the world,” he had acted, in all respects, as a brake on animal *développement*. His disruptive influence—by altering natural balances, hunting, transferring species to unsuitable regions, and destroying communities—had hindered animal life and the exercise of those functions that could produce a greater perfection. The gap between man, apes, and other animals was thus destined to grow ever wider.

Confirming his interest in social issues, Bonelli compared these phenomena to what happened when an ideal republican order was subverted. Whenever an individual favored by circumstances was able to prevail over others, his superiority was destined to increase with time. A typical case was that of the founder of a dynasty: after initially acting as *primus inter pares* [first among equals], he then proceeded to gain more and more control over the rest of his fellow citizens, thanks to his weapons or talent, stifling their ambitions to challenge his authority.^61^

When he was younger, Bonelli had believed in a harmonious model of the economy of nature, in which man, animals, plants, and minerals occupied a necessary and mutually useful place, preserving an ecological balance that testified to the wisdom of nature. Now that picture appeared to be called into question by man himself, the most outstanding living being on the physical and moral level. Bonelli was clearly recalling some of the passages from *Philosophie zoologique*, where Lamarck had sharply underlined man’s part in the destruction of the ecosystem (1809, vol. 1, pp. 351–352). In his work, Lamarck bluntly stated that man was nothing more than a perfected quadruman, thus admitting a close relationship between man and apes. Bonelli, on the other hand, despite approaching a similar position, never took it to its extreme consequences. He avoided confronting the issue directly on several occasions, instead vigorously claiming a form of uniqueness for the human species.

## Finding a Solution: Transformism Applied to Anthropology

By situating man within the framework of the process of transformation and improvement common to all living beings, Bonelli’s handwritten papers suggest the hypothesis of a relationship. As a result both of his religious beliefs and the social implications he had raised in his letter to Ziegler, he was unable to take the final step along this dangerous path. In 1818, during the Restoration, he spoke bluntly during his university lectures: despite the lack of human fossil remains, man was “an ancient product of nature, according to a plan established since the origin of the world, towards which every other being of later formation still tends, without ever being able to reach it.” Bonelli thus reaffirmed the gap between man and apes, which had become unbridgeable because some characters had not evolved at the right time. Moreover, man was the most perfect being, “the only one made in the likeness of God: the ape is just the summit of the animal branch directed towards man, modeled on the plan of his organization, such that I shall never take the view that the origin of man can be attributed to a perfected ape.”^62^

Despite some hesitations, doubts, and reversals, however, Bonelli saw the natural history of man as a decisive tool in the defense of transformism. In the conclusion to his letter to Ziegler and elsewhere in his papers, Bonelli presented man as an example of the inconsistency that reigned among classifications. Naturalists considered the differences between human varieties to be negligible, while they employed much stricter criteria to evaluate animal characters and to create new species based on modifications that could be reduced to simple intraspecific variations. Thanks to this repeated observation, during the Restoration period Bonelli developed an argument to respond to the accusations of atheism and betrayal of the Holy Texts that had been tormenting him for a long time.

After 1814, the reinstatement of the Savoy monarchy and the Church’s interference in every sector of society encouraged Bonelli to seek conciliation between transformism and religion. Such concerns are evident in some of his writings that are dedicated to the problem of the “overflowing multiplication of species in natural history.” Reflecting on a taxonomic system that could replace the chain of beings, Bonelli suggested the image of an intricate ramification. Such a structure raised a further question: should branch bifurcations be considered as unchanging or should a diachronic factor be introduced that would give genealogical depth to an ever-changing tree? The answer was obviously the latter and, to justify this idea, Bonelli called man into question.

Fixism, along with strict compliance to traditional taxonomic methods, presented a “very serious inconvenience, disreputable to the decorum of the common origin of the human species transmitted to us by the Holy Scriptures,” because it could lead to an endorsement of polygenic theories^63^:

Physically speaking, the differences between the main varieties of man, and often also between subordinate ones, are of much greater consequence and importance than those that occur between one animal and another, one plant and another, belonging to what are usually called true species.^64^

To safeguard the unity of the human species since its outset, as handed down in the Book of Genesis, the only solution was to accept in all beings “a certain variability, inconstancy, versatility, tendency to change or to vary in consequence and direction of the influence of physical circumstances.” In the case of man, Bonelli examined this variability in intraspecific terms, but he had at times considered it from such a perspective as to configure a genealogical link with other animals. Nature’s predisposition to variation was necessarily unlimited—since “the Supreme Creator” had placed no limit on his “double command, *crescite et multiplicamini*”—and this was therefore the basic premise underpinning any transformist explanation.

Bonelli believed his interpretation of that divine order was completely legitimate because the Scriptures specified neither the number nor the qualities of the animals at the time of creation. However, since they had appeared before Adam, their creation should have occurred in a circumscribed setting and organisms should have been in harmony with local circumstances. God’s command explained their migration instinct and the subsequent multiplication of their species. As such, original creation already included the guidelines for subsequent development, according to a course laid out by the Supreme Wisdom.

Such a solution, Bonelli noted, was “in line with Christian belief.” It could satisfy both religion and science and answer questions that, if left ambiguous, would have compromised the solidity of morals.^65^ Transformism thus drew its strength and *raison d’être* precisely from the issue of man, which, instead of being the greatest obstacle, became its backbone.

Bonelli’s interest in the history of species—the hallmark of his entire activity—thus went hand in hand with sensitivity to anthropological issues still uncommon in Italy at the time. In both cases, his reflections did not circulate outside the peripheral milieu of Turin, and often even within it, and they failed to have any significant impact on the scientific community. After Bonelli’s death, Lamarckian research, except for some scholars who worked on it privately, suffered a phase of stagnation, if not of genuine ostracism, even in Piedmont, only to reappear in 1848.^66^ The situation was no better for anthropology. When, in 1857, Giustiniano Nicolucci (1819–1904), the Italian founder of the discipline, published *Delle razze umane* [On the Human Races] (1857–58), he regretted the lack of consideration accorded to those studies in the peninsula. The questions Bonelli had asked himself almost half a century before would, however, remain highly relevant for a long time to come.

## Footnotes

1 Bonelli’s activities are dealt with extensively in Forgione (2018, pp. 17–151), where they are placed in the scientific and political context of nineteenth-century Piedmont.

2 See Camerano (1910, pp. 418–420) and Baccetti and Omodeo (1969).

3 An idea of Bonelli’s readings comes from references scattered in his notebooks and the recommendations mentioned during his courses of lectures. These papers are held in the Bonelli Fonds (hereafter cited as FB), at the Historical Library of the Department of Life Sciences and Systems Biology of the University of Turin – Section of Animal and Human Biology (hereafter cited as Dbios). See, in particular, *Bibliothèque zoologique*, in Dbios, FB, Faldone X, *Corso di Generalità Zoologiche. Compito. Dato in francese negli anni 1811–1813*.

4 Spinola Archive, Tassarolo, Italy, Letter from Franco Andrea Bonelli, 23 April 1808, n. 86: “Quelle differenze che al solo clima o nodrimento sono dovute, e che per conseguenza non dovrebbero entrare nei caratteri specifici, furono appunto quelle su cui si fondarono tante e tante specie che col nome di razze o di varietà costanti nella data specie si potrebbero indicare.” Throughout the article, I will provide the original passages in footnotes that refer to the English translations and paraphrases provided in the text.

5 Dbios, FB, Faldone 1, *Part 21 des Mémoires ornithologiques*, n. 1855: “Les organes semblent destinés à la façon de vivre, et non la façon de vivre aux organes, nous avons une preuve dans les canards. Le sauvage qui passe sa vie dans les lacs, et les rivières a les pattes comprimées comme dans tous les oiseaux nageurs, le domestique au contraire qui contraint dans une basse court, où à peine trouve-t-il un peu d’eau, mais pas assez pour y nager, a les pattes presque rondes. Les autres parties telles que les ailes, les membranes ec. doivent aussi subir des changements. Rarement voit-on un canard domestique à voler.”

6 On ornithological examples in contemporary works—including the Sonnini edition of Buffon’s *Histoire naturelle*, annotated by Virey—and on Virey’s opposition to Lamarck, see Corsi (1987; 2001, pp. 121–129, 205–219) and Burkhardt (1995, pp. xxiii–xxxiii). Lamarck (1802, p. 50) affirmed: “Ce ne sont pas les organes, c’est-à-dire la nature et la forme des parties du corps d’un animal, qui ont donné lieu à ses habitudes et à ses facultés particulières; mais ce sont au contraire ses habitudes, sa manière de vivre, et les circonstances dans lesquelles se sont rencontrés les individus dont il provient, qui ont avec le temps constitué la forme de son corps, le nombre et l’état de ses organes, enfin les facultés dont il jouit.” Virey (1803a, p. 411) replied: “Ce n’est donc point la plante, l’animal qui donnent lieu à leur conformation par leurs habitudes, puisque ces habitudes sont déterminées par leur configuration organique. En effet, l’oiseau ne pouvoit pas se donner l’habitude de s’élever dans les airs, s’il n’avoit pas reçu des ailes …. On auroit donc tort de prétendre que c’est l’habitude qui a présidé à la formation de tous les êtres, puisque cette habitude n’en est que le résultat.” Despite his encyclopedic attitude, which led him to sum up doctrines seemingly in contradiction with each other, Virey repeatedly championed vitalism and providentialism; see Rey (1988) and Corsi (2018).

7 See Dbios, FB, Faldone 1, *Parte 16*^*a*^* delle Memorie Zoologiche*, n. 1226.

8 For this definition, see Ricuperati (1994). Outram (1976) and Boudard (1988) offer an overview of the fortunes of the University of Turin during the French years. Detailed insights are also provided by Romagnani (1988–1990), a work entirely dedicated to the activities of Prospero Balbo, who headed the Piedmontese educational system for a long period of time. For the final report of Cuvier’s Italian tour, see Cuvier et al. (1810).

9 On his meetings with Lamarck, see the letter that Bonelli sent to his relatives on 21 September 1810, held at the Civic Museum of Cuneo: “J’ai fait au jour d’hui une visite à M. Lamarck qui m’ayant trouvé partisan de quelques unes de ses idées me prit en amitié, m’instruisit sur bien de choses et m’accorda des grandes facilités pour étudier les animaux invertébrés, puisque cette partie du Muséum est sous sa direction.” Bonelli had already mentioned Lamarck in one of his entomological manuscripts, while analyzing examples of refinement and deterioration in insects; see Dbios, FB, Faldone 3, Sugli insetti e sugli aracnidi, *Observations entomologiques, 1*^*ère*^* partie, 2*^*nd*^* mémoire (continuation de l’avant-propos)*.

10 For a list of Lamarck’s pupils, see the online census edited by Pietro Corsi at https://www.lamarck.cnrs.fr, also available at https://redi.imss.fi.it/lamarck.

11 See the draft of a letter from Bonelli to Ziegler dated 5 May 1806, in Dbios, FB, Faldone 2, *Miscellanea mea, Cahjer n. 1, Mémoires du jour*. Like Bonelli, Ziegler did not publish his naturalistic research, but his name is attached to many insect species. When he retired in 1823, he devoted himself more extensively to malacology and to land and freshwater mollusks. For biographical information on Ziegler, see Schmidt (1846), Fitzinger (1868, pp. 1042–1047, 1089), Staut-Turk (1983), and Bögel (2013).

12 The handwritten draft of Bonelli’s letter is currently unavailable, but the text was published in Camerano (1908a).

13 Bonelli to Ziegler, in Camerano (1908a, p. 5): “Il Signor Lamarck nella sua *Phylosophie zoologique* ha posto (dopo però molte osservazioni che appartengono a Pallas, Buffon e molti altri zoologi osservatori) i germi e molte prove indirette in favore di tal dottrina, ma se lo stesso autore fosse stato meno pensatore e più minuto osservatore, e sopratutto se fosse disceso negli ultimi detagli di qualche porzione di Zoologia ed avesse studiato un più gran numero d’animali, avrebbe tirato un partito infinitamente migliore delle sue viste, ed avrebbe in qualche modo evitato degli errori e molte considerazioni ridicole che fan un grandissimo torto alle osservazioni e considerazioni fondate, colle quali sono mescolate.”

14 Lamarck used the term *perfection* to describe organic complexity, while remaining convinced that any animal could be considered to be perfect if its organization met its life needs. See Lamarck (1815–22, vol. 1, p. 139): “Tout corps vivant, quel qu’il soit, est un être réellement parfait, c’est-à-dire, un être à qui il ne manque rien de ce qui lui est nécessaire! mais, la nature ayant composé de plus en plus l’organisation animale; et par là, étant parvenue à douer ceux des animaux qui possèdent l’organisation la plus compliquée, de facultés plus nombreuses et plus éminentes, on peut voir dans ce terme de ses efforts, une perfection dont s’éloignent graduellement les animaux qui ne l’ont pas obtenue.” Bonelli defined *perfection* as “the structure that is the most eminently harmonious with habits and in which the organization looks perfect, i.e. in the maximum development of the most common and normal necessary parts and in the absolute disappearance of the useless or transitory ones.” See *Perfezione*, in Camerano (1906, p. 6).

15 Bonelli to Ziegler, in Camerano (1908a, pp. 6–7): “A guisa d’una sfera d’orologio che faccia il suo giro in mille anni e di cui perciò il moto non divien a noi visibile e sensibile che per il paragone fatto in tempi distanti.”

16 Lamarck (1809, vol. 1, p. 132). For a discussion, see Barsanti (1976, 1979, pp. 198–208); Burkhardt (1995, pp. 144–164); Corsi (2001, pp. 163–179, 235–248).

17 Lamarck (1809, vol. 1, p. 374): “Le propre du mouvement des fluides dans les parties souples des corps vivans qui les contiennent, est de s’y frayer des routes, des lieux de dépôt et des issues; d’y créer des canaux, et par suite des organes divers; d’y varier ces canaux et ces organes à raison de la diversité, soit des mouvemens, soit de la nature des fluides qui y donnent lieu et qui s’y modifient; enfin, d’agrandir, d’allonger, de diviser et de solidifier graduellement ces canaux et ces organes.”

18 Lamarck (1809, vol. 2, p. 54): “Ce mouvement des fluides a … la faculté de composer peu à peu l’organisation, en multipliant les organes et les fonctions à remplir, à mesure que de nouvelles circonstances dans la manière de vivre, ou que de nouvelles habitudes contractées par les individus, l’excitent diversement, exigent de nouvelles fonctions, et conséquemment de nouveaux organes.”

19 Lamarck (1802, pp. 48–49, 65): “En partant du plus simple pour s’élever graduellement jusqu’aux objets les plus composés, qui est-ce qui ne voit pas dans les faits que je viens de citer les résultats très-marqués de la tendance du mouvement organique, à développer et composer l’organisation, et en même temps celle qu’il a à réduire en fonctions particulières à certaines parties … des facultés générales et communes à tous les points du corps de l’individu? … L’usage de la vie tend à développer l’organisation, et même à composer et à multiplier les organes, comme le prouve l’état d’un animal qui vient de naître, comparé à celui où il se trouve lorsqu’il a atteint le terme où ses organes (commençant à se détériorer) cessent d’exécuter de nouveaux développemens.”

20 See Dbios, FB, Faldone X, *Corso di generalità zoologiche. Compito. Dato in francese negli anni 1811–1813, Influence des circonstances environnantes*, where Bonelli defined it as a “marche … indépendante, et peut-être casuelle (volonté de dieu),” or *(Plan de mon) Crescite et multiplicamini, ou Tableau généalogique du Règne animal (Février de l’an 1814)*, in Camerano (1908b, p. 30): “Impression que la nature (Dieu) a donnée [aux animaux] (force incompréhensible et inexplicable qui les y fait tendre comme elle fait tendre tous les corps au centre).”.

21 Lamarck (1809, vol. 1, p. 107): “Cette échelle … n’offre des degrés saisissables que dans les masses principales de la série générale, et non dans les espèces, ni même dans les genres: la raison de cette particularité vient de ce que l’extrême diversité des circonstances … fait naître dans les formes et les caractères extérieurs, des anomalies ou des espèces d’écarts que la composition croissante de l’organisation n’auroit pu seule occasionner.”

22 Dbios, FB, Faldone X, *Corso di generalità zoologiche. Compito. Dato in francese negli anni 1811–1813, Influence des circonstances environnantes*.

23 Corsi (2001, pp. 235–246) believes that Lamarck’s insistence on the idea of *pouvoir de la vie* was due to his inability to explain, in detail, each step of the series of living beings through the movement of organic fluids.

24 See *Mouvement et Marches de la nature vivante*, in Camerano (1908b, pp. 36–38), and *Sulla soverchia moltiplicazione delle specie in storia naturale e Sull’Unità del Genere umano**, **provata colla incostanza o variabilità degli animali e questa coll’osservazione e colle parole della Genesi*, in Camerano (1909b, pp. 4–5). Bonelli was proud of his explanation, so much so that he planned to publish a work entitled *Crescite e multiplicamini*; see *(Plan de mon) Crescite et multiplicamini**, **ou Tableau généalogique du Règne animal (Février de l’an 1814)*, in Camerano (1908b, pp. 29–36).

25 “Cosa altrettanto indegna di un uomo ragionevole, quanto lo sarebbe di voler ancor darsi ad intendere che il sole gira attorno alla terra, che *tutti* gli animali *indistintamente* siansi trovati nell’Arca di Noè, ed altre simili puerili, inconseguenti e scioccamente immaginate filastrocche che possonsi credere dai ciechi nati o da chi non ha il senso comune, o da chi almeno non ne vuol fare uso.” In 1838, Darwin expressed a similar position in his *Notebook D*, rejecting the idea that God had specifically created “the Rhinoceros of Java and Sumatra” and “a long succession of vile molluscous animals.” See Barrett et al., eds. (1987, p. 343).

26 “L’uomo per esempio, quivi è bianco, in Africa è nero, nella Lapponia è basso, nella Patagonia è alto etc. tutte queste differenze son bagatelle. L’uomo è un solo dapertutto, e mentre così largamente giudichiamo, con una minutezza e una severità affatto apposte, contiamo il numero dei punti della coccinella *24 punctata* e nella *Tinea evonimella*, osserviamo le diverse più fugaci tinte di un papilione, separiamo il *lucanus capreolus* dal *cervus*, distinguiamo il lepre dal coniglio, la *cicindela danubialis* dall’*hibryda* etc. etc.”

27 Among the extensive scholarship on the controversial boundaries between man and apes, see Barsanti (1983, 1990, 2009), Blanckaert (2014), Corbey and Theunissen, eds. (1995), Corbey (2005), Sebastiani (2015, 2019), and Tinland (2003).

28 This issue is pointed out by Douglas (2008, pp. 53–58), who reconstructs the history of racial studies until the Darwinian synthesis, focusing on the interweaving of environment and heredity as explanatory factors of human differences.

29 See *Praefatio*, in Linné (1746). Nevertheless, Linnaeus recognized a radical difference between them in terms of intelligence and reasoning.

30 See Linné (1758, pp. 18–24). On the importance for the emergence of the concept of race of the shift from logical-classificatory thinking to genealogical thinking, see Doron (2016, pp. 419–449).

31 Buffon (1749, p. 437): “Lorsque nous mettons l’homme dans l’une de ces classes [des animaux], nous ne changeons pas la réalité de son être, nous ne dérogeons point à sa noblesse, nous n’altérons pas sa condition, enfin nous n’ôtons rien à la supériorité de la nature humaine sur celle des brutes, nous ne faisons que placer l’homme avec ce qui lui ressemble le plus, en donnant même à la partie matérielle de son être le premier rang.”

32 Thus, within the natural history of man, the genealogical definition of race, connected to the transmission of variations, placed the concept of species under stress. On Buffon’s anthropological ideas and his concept of race, see the different positions of Blanckaert (2003) and Doron (2016, pp. 450–487). Unlike Blanckaert, Doron argues that Buffon was the first naturalist to theorize a genealogical concept of race.

33 See *De hominis a ceteris animalibus differentia*, in Blumenbach (1795, pp. 1–64). Blumenbach’s work was published in 1775 as a doctoral dissertation, but its third and most influential edition appeared in 1795.

34 See *Generis humani varietates quinae principes, species vero unica*, in Blumenbach (1795, pp. 284–322).

35 On the notion of *Bildungstrieb* and the relationship between Kant and Blumenbach’s ideas on human races, see Lenoir (1980, 1982, pp. 17–35), Bertoletti (1990, 1994), and Richards (2000, 2002).

36 Camper’s fundamental work, published posthumously, is Camper (1791). On his anthropologic ideas, see Meijer (1999); on the use of the facial angle and craniometry during the nineteenth century, see Blanckaert (1987) and Doron (2016, pp. 371–383). On the context in which Camper’s activities took place, see Van Berkel and Ramakers, eds. (2015).

37 See Camper (1782).

38 Cuvier and Saint-Hilaire (1795, p. 457): “On observe dans les diverses races d’hommes la même série de rapports, que dans les diverses espèces d’animaux, entre la saillie du crâne et le degré d’intelligence…. Nous ne voyons pas du moins qu’aucun des peuples à front déprimé et à mâchoires proéminentes, ait jamais fourni de sujets égaux au général des Européens par les facultés de l’âme;” Cuvier (1800, pp. 2–15): “La proportion respective du crâne et de la face indiquant immédiatement celle du cerveau, avec deux des principaux organes extérieurs, est donc aussi un indice du plus ou moins de perfection des facultés intérieures comparées avec les extérieures.” On the overcoming of the dualism between *homme physique* and *homme moral* and on the passage from the natural history of man to a properly anthropological science, see Barsanti (1983, pp. 128–195).

39 Cuvier (1817, pp. 94, 95): “Il y a cependant des causes intrinsèques qui paraissent arrêter les progrès de certaines races, même au milieu des circonstances les plus favorables…. Son [de la race nègre] museau saillant et ses grosses lèvres, la rapprochent manifestement des singes: les peuplades qui la composent sont toujours restées barbares.”

40 See, in particular, Dbios, FB, Faldone 5, *Cours complet sur les mammifères* and *Histoire de l’homme*. Bonelli also hinted at the classifications proposed by other authors, who were the same as those cited by Blumenbach in his work.

41 On the role played by skin color in shaping physical anthropology, see Mazzolini (2014).

42 *Elementi di zoologia per l’anno 1818*, in Camerano (1909a, pp. 15–16): “Giacché il principio di prender per tippo l’uomo di quella sottospecie che [appare] più bella e più regolare nelle sue forme e proporzioni a sola competenza del giudicio nostro, è poco ragionevole.”

43 Among other entries, see Virey (1803b, 1817). On the competitive market of scientific dictionaries in France, see Corsi (2012). On Virey’s anthropological tenets, see Barsanti (1983, pp. 149–169) and Blanckaert (1988).

44 Virey (1801, p. 423): “L’angle facial … donne, pour ainsi dire, la mesure du rapport entre la pensée et l’appétit physique.” At the turn of the century, Virey (1801, p. 138) had not yet fully developed a classification into distinct species. Later he had no doubts: “Le genre humain, dans sa totalité, doit se diviser en deux espèces distinctes, et celles-ci se partagent ensuite en diverses races ou souches principales et en familles” (1817, p. 152). Virey (1801, p. 412; 1817, p. 150) rejected interbreeding as a criterion to distinguish species. See also White (1799).

45 Virey (1801, p. 428): “Croyez-vous qu’il y ait si loin des hottentots boschmanns, au chimpanzée qui habite presque dans le même pays? Ce museau triangulaire, cet aspect ignoble et louche, cette intolérable grossièreté de la voix et des manières, cette excessive étroitesse de l’entendement, ces habitudes toutes animales, toutes concentrées dans les appétits physiques, tout dénonce leur stupide animalité et leur excessive hébétation …. Certainement il y a plus de distance de ceux-ci à Newton, à Montesquieu, à Buffon, etc., que des singes à ces peuples stupides;” Virey (1817, p. 18): “Le nègre est adonné en général à ses sens matériels, et il a toujours montré moins d’industrie et de facultés intellectuelles que l’homme blanc.” Virey defined man as the only two-handed and bipedal animal and distinguished his species and races. However, he observed a gradual variation of anatomical characters, connecting white man to black man and apes. On this and other ambiguities, see Blanckaert (1988). For an overview of Virey’s activity, see Bénichou and Blanckaert, eds. (1988).

46 Bory de Saint-Vincent (1825, p. 340): “L’Homme intellectuel … n’est qu’une conséquence nécessaire de l’Homme mammifère.” See also Bory de Saint-Vincent (1827) and, on his life and works, Ferrière (2009).

47 Bory de Saint-Vincent (1825, p. 269): “On doit adjoindre [à l’ordre des bimanes], pour le rendre complètement naturel, le genre Orang.” Daubenton’s theories are in Daubenton (1764, 1782).

48 See Bory de Saint-Vincent (1825, p. 277) and Doron (2016, pp. 437–438).

49 See Bory de Saint-Vincent (1825, pp. 276–336). However, Bory pointed out that, “si la supériorité intellectuelle de quelques Hommes favorisés sortis de l’espèce Japétique, paraît mériter à celle-ci le premier rang, les neuf dixièmes des individus qui la composent ne sont cependant pas beaucoup supérieurs aux Hottentots, quant au développement de la raison. Nous n’avons donc la prétention d’assigner aucune place définitive” [if the intellectual superiority of some favored men of the Japhetic species seems to earn to this one the first rank, however nine-tenths of the individuals which compose it are not much superior to the Hottentots, as for the development of reason. Therefore, we do not presume to assign any permanent place] (1825, p. 328).

50 “L’homme forme à lui seul une espèce, un genre, un ordre isolé et distinct de tous les autres non seulement par ses facultés morales, mais aussi par des caractères physiques pris de la conformation de ses parties les quelles présentent l’ensemble le plus complet, le plus harmonique, le plus univoque, enfin le plus parfait sous tous les rapports.”

51 For this passage, see *Conclusion de l’ouvrage*, in Camerano (1908b, p. 36).

52 Dbios, FB, Faldone 5, *Cours complet sur les mammifères*: “On s’est efforcé de nos temps de chercher les rapports qui rendent l’espèce du singe si semblable à celle de l’homme, de trouver l’analogie, en un mot de justifier une comparaison que l’on voulait établir entre l’organisation et les facultés de l’un et celles de l’autre. Cette idée, très ancienne d’ailleurs, et qui tendait peut être moins à élever l’homme au rang que l’auteur de la nature lui assigna, qu’à le dégrader en élevant à son rang des animaux anthropomorphes, des caricatures grossières de sa figure, cette idée dis-je fixa l’attention sur plusieurs caractères sur les quels on n’avait pas encore porté l’attention, mais qui furent par la suite reconnus de telle importance, que même sans ces 2 facultés sublimes, sans le raisonnement et la parole, l’homme seroit encore éminemment distingué et placé à un rang infiniment et sans aucun comparaison plus élevé que les singes par la seule considération de son physique.”

53 Dbios, FB, Faldone 1, *Libro 14 delle memorie zoologiche*, n. 1098: “Je trouve, que les Naturalistes ont pris des caractères trop douteux pour distinguer l’homme d’avec les Brutes. Ils ont dit *l’homme pense, raisonne, parle*. Tout cela ne vaut pas riens. En primiere lieu *homme pense*. Que c’est il *penser*? C’est il une faculté particulière à l’homme? C’est il une faculté dipendente de la puissance sur tous les autres animaux?”

54 Lamarck (1802, pp. 124–140): “On observe une sorte de gradation dans l’intelligence des animaux, comme il en existe une dans le perfectionnement de leur organisation, et on remarque qu’ils ont des idées, de la mémoire, qu’ils pensent, qu’ils choisissent, qu’ils aiment, qu’ils haïssent, qu’ils sont susceptibles de jalousie, et que par diverses inflexions de leur voix et par des signes, ils se communiquent et s’entendent …. Je me suis apperçu que celui de tous les organes du corps de l’homme … en qui les effets de l’exercice et d’une habitude d’emploi sont les plus considérables, c’est l’organe de la pensée, en un mot, c’est le cerveau de l’homme.”

55 Lamarck (1802, p. 134): “Ne pourroit-on pas penser que cet état particulier de l’organisation de l’homme a été acquis peu à peu à la suite de beaucoup de temps, à l’aide des circonstances qui s’y sont trouvées favorables?” See also Corsi (2001, pp. 180–183).

56 Lamarck (1809, vol. 1, p. 349): “Si une race quelconque de quadrumanes, surtout la plus perfectionnée d’entre elles, perdoit, par la nécessité des circonstances, ou par quelqu’autre cause, l’habitude de grimper sur les arbres, et d’en empoigner les branches avec les pieds, comme avec les mains, pour s’y accrocher; et si les individus de cette race, pendant une suite de générations, étoient forcés de ne se servir de leurs pieds que pour marcher, et cessoient d’employer leurs mains comme des pieds; il n’est pas douteux … que ces quadrumanes ne fussent à la fin transformés en bimanes, et que les pouces de leurs pieds ne cessassent d’être écartés des doigts, ces pieds ne leur servant plus qu’à marcher.”

57 Lamarck (1809, vol. 1, pp. 349–357, and vol. 2, pp. 346–387). See Corsi (2001, pp. 251–252) and Stoczkowski (1997, pp. 458–462). See also Lamarck (1820, p. 149): “L’homme, véritable produit de la nature, terme absolu de tout ce qu’elle a pu faire exister de plus éminent sur notre globe, est un corps vivant qui fait partie du règne animal, appartient à la classe des mammifères, et tient par ses rapports aux quadrumanes dont il est distingué par diverses modifications, tant dans sa taille, sa forme, sa stature, que dans son organisation intérieure; modifications qu’il doit aux habitudes qu’il a prises et à sa supériorité qui l’a rendu dominant sur tous les êtres de ce globe.” The question of man was also addressed in an entry for the *Nouveau dictionnaire d’histoire naturelle*; see Lamarck (1817).

58 See *(Plan de mon) Crescite et multiplicamini, ou Tableau généalogique du Règne animal (Février de l’an 1814)*, in Camerano (1908b, pp. 32–33) and Forgione (2018, pp. 57–58). On the interplay between anthropology and social theories, see Barsanti (1983, pp. 177–195) and Blanckaert (2000).

59 *(Plan de mon) Crescite et multiplicamini, ou Tableau généalogique du Règne animal (Février de l’an 1814)*, *Conclusion de la I*^*ère*^* partie*, in Camerano (1908b, p. 33): “En bonne phylosophie on ne peut admettre une création particulière pour l’homme, ni des conditions étrangères à celles qui ont suivi la formation des autres animaux inférieurs à lui.”

60 Bonelli in Camerano (1908b, p. 34): “L’homme vraiment phylosophe et impartial à son propre égard dans le jugement qu’il porte sur la masse entière des animaux, doit reconnaitre que lui seul, comparativement à l’univers dont il se croit le maitre ou tout au moins l’agent et l’objet principal, n’est absolument que poussière comme le dit l’écriture Sainte, et que c’est son orgueil seul qui a pu la lui faire croire, étant constaté actuellement par la géologie, la zoologie, et les connaissances que l’on a sur les fossiles, que le globe a existé longtens avant lui et que tous les animaux fossiles même sont de formation antérieure à la sienne, puisque nulle part il se trouve fossile.” Bonelli echoed Cuvier’s ideas against the existence of the fossil man; on the debate on the antiquity of man in France, see Laurent (1989).

61 See *Multiplication successive des espèces sauvages d’animaux. Perfectionnement et Unité de l’espèce humaine*, in Camerano (1908b, pp. 41–44).

62 See *Elementi di zoologia per l’anno 1818*, in Camerano (1909a, pp. 14–15). On Bonelli’s cautions during the Restoration, see Forgione (2018, pp. 106–151).

63 *Sulle conseguenze della soverchia moltiplicazione delle specie in storia naturale e Sull’Unità del Genere umano**, **provata colla incostanza o variabilità degli animali e questa coll’osservazione e colle parole della Genesi*, in Camerano (1909b, p. 3): “Si cade con questa ipotesi in un gravissimo inconveniente disdicevole al decoro della comune origine trasmessaci dalle S. Carte, della specie umana.” Conciliatory attempts were also made among polygenists, as proven by Bory de Saint-Vincent. According to him, the Genesis only referred to Jewish history, and thus dealt with the very first man, not to be considered as the ancestor of the whole of humanity, but rather as the founder of his own species. See Bory de Saint-Vincent (1825, p. 277).

64 Bonelli in Camerano (1909b, p. 3): “Fisicamente parlando, le differenze che passano fra le principali, e sovente anche tra le varietà subalterne d’uomini, sono diffatti ben più conseguenti e importanti che non quelle che passano tra un animale ed un altro, una pianta ed un’altra, di quelle alle quali si attribuisce, nel uso, il nome di vera specie.”

65 See Bonelli in Camerano (1909b, p. 3): “Un’opinione che credo di tutta certezza nella sua essenza, conforme alla credenza cristiana.”

66 Bonelli’s successor, Giuseppe Gené (1800–1847), strongly opposed transformism, which he believed to be the result of the revolutionary and Napoleonic upheavals. On Gené and the history of evolutionary studies in Turin, see Corsi (1983) and Forgione (2018).
